# Safety of liposomal daunorubicin-cytarabine (CPX-351) in secondary AML: Japanese phase 1/2 study and global phase 3 study

**DOI:** 10.1007/s12185-025-04047-4

**Published:** 2025-08-15

**Authors:** Naoko Hosono, Yusaku Tomiyama, Nanako Emori, Kento Isogaya, Takahiro Yamauchi

**Affiliations:** 1 Department of Hematology and Oncology, Faculty of Medical Sciences, University of Fukui, 23-3 Matsuoka Shimoaizuki, Eiheiji-cho, Yoshida-gun, Fukui, 910-1193 Japan; 2 Medical Planning Department, Nippon Shinyaku Co., Ltd, Kyoto, Japan; 3 Data Science Department, Nippon Shinyaku Co., Ltd, Kyoto, Japan

**Keywords:** Acute myeloid leukemia, CPX-351, Liposomal cytarabine, Liposomal daunorubicin, Secondary AML

## Abstract

CPX-351 is a dual-drug liposomal encapsulation of cytarabine and daunorubicin. The safety of CPX-351 in Japanese patients was verified by comparing safety data from the Japanese phase 1/2 study and the global phase 3 study. This analysis included 47 patients in the safety analysis set of the Japanese study and 153 patients who received CPX-351 and were included in the intention-to-treat analysis for the global study. In both the Japanese and global studies, the most frequent adverse events were febrile neutropenia (FN) (Japanese: 85.1%, global: 70.0%) and pneumonia (25.5% and 24.2%). Median time to occurrence was 8 and 11 days for FN, and 17 and 23 days for pneumonia. Median time to hematologic recovery was 36 and 41 days for neutrophils (> 1000/μL), and 36 and 44 days for platelets (> 100,000/μL). In the global study, univariate analysis was performed after one cycle of induction therapy to investigate factors that delay neutrophil and platelet recovery, but no specific factors were identified. In these analyses, the pattern and frequency of adverse events were similar in Japanese and non-Japanese patients, with no need for race- or region-specific management.

**Supplementary Information:**

The online version contains supplementary material available at 10.1007/s12185-025-04047-4.

## Introduction

Acute myeloid leukemia (AML) are highly diverse heterogenous hematologic malignancies characterized by clonal autonomous growth of myeloid precursor cells with impaired differentiation and maturation. As a result of the abnormal proliferation of leukemic cells in the bone marrow, normal hematopoietic function is severely impaired, resulting in a variety of symptoms associated with leukopenia, anemia, and thrombocytopenia. Without appropriate treatment, this serious disease can lead to death in a short period of time due to infection or bleeding [1–3]. In particular, secondary AML, such as AML with myelodysplasia-related changes (AML-MRC), which develop from myeloid malignancies such as myelodysplastic syndromes (MDS) and chronic myelomonocytic leukemia (CMML), and therapy-related AML (t-AML) have a poorer prognosis compared to de novo AML [4–7].

CPX-351 is a dual-drug liposomal encapsulation of cytarabine and daunorubicin at a fixed 5:1 synergistic molar ratio [8, 9], has demonstrated better efficacy than the 3 + 7 treatment for newly diagnosed secondary AML patients in the pivotal, randomized controlled study [10]. It has been available in U.S. since 2017, in Europe since 2018, and in Japan since 2024. CPX-351 delivers cytarabine and daunorubicin to leukemia cells while maintaining a synergistic 5:1 molar ratio, which is not only highly cytotoxic but also highly effective due to its sustained retention in the bone marrow [8, 9]. However, its long-term action in the bone marrow results in a delay in blood cell recovery. In fact, the pivotal study has shown that the time to neutrophils and platelets recovery in the CPX-351 group was delayed by about 1 week compared to conventional 3 + 7 group [10]. On the other hand, the safety characteristics of CPX-351 have shown that the pattern and frequency of adverse events are similar to those of 3 + 7, except for the delayed recovery from myelosuppression [10]. The adverse event (AE) profile was similar in the Japanese phase 1/2 study [11] and some real-world data (RWDs) [12–14], although there is some variation in the frequency of AEs.

While there is variable information on the frequency of adverse events during CPX-351 treatment, there are no reports detailing when the most common adverse events occur or how they are managed. Therefore, we investigated potential differences in the frequency of AEs and the management of AEs during CPX-351 treatment in the Japanese P1/2 study and the global phase 3 study.

## Materials and methods

### Patients, eligibility criteria, and study oversight

This analysis included 153 patients who participated in the global P3 from December 2012 to November 2014 and 47 patients who participated in the Japanese P1/2 study from August 2019 to October 2021.

Japanese P1/2 and global P3 studies eligibility criteria have been described in detail elsewhere [10, 11]. Patients were enrolled in the Japanese P1/2 study and the global P3 study who received CPX-351.

Each study protocols were approved by the independent ethic committee or institutional review board at each study site and each study was conducted according to the principles of the Declaration of Helsinki, International Conference on Harmonization Good Clinical Practice guidelines and Good Clinical Practice for Drugs. All patients provided written informed consent.

### Study design and treatment

Each study design was described previously [10, 11]. The Japanese P1/2 study was open-label, single arm study and the global P3 study was open-label study randomly assigned patients to receive CPX-351 or conventional cytarabine and daunorubicin. The initial CPX-351 induction course consisted of 100 units/m^2^ (100 mg/m^2^ cytarabine and 44 mg/m^2^ daunorubicin) administered as a 90-min infusion on days 1, 3, and 5. A second induction course (same dose) was administered on days 1 and 3 for patients who did not achieve hypoplastic marrow on a day 14 (global study) or 15 (Japanese study) bone marrow assessment. For patients in CR/CRi after induction, postremission therapy consisted of up to two cycles of 65 units/m^2^ CPX-351 (65 mg/m^2^ cytarabine and 29 mg/m^2^ daunorubicin) on days 1 and 3.

### Endpoints and assessments

The primary study endpoint in the Japanese P1/2 study was the rate of composite complete remission (CRc:CR + CRi) at the end of the induction cycles and that in global P3 study was overall survival (OS), those were also described previously [10, 11].

In this safety analysis, patients enrolled in the Japanese P1/2 study and the global P3 study who were administered CPX-351 were included.

The method of content newly described in this study is as follows.

1. Days to neutrophil and platelet recovery

The distribution of days to neutrophils or platelets recovery was estimated using the Kaplan–Meier method, and the median and first and third quartiles are shown. The number of days to neutrophils or platelets recovery was defined as follows: (i) The start date is the date of the beginning of the remission induction therapy. In patients who have received twice induction therapy, the start date is the date of the last induction remission therapy. (ii) The date of recovery is defined as the date on which the platelet count (≥ 50,000/µL or ≥ 100,000/µL) or neutrophil count (≥ 500/µL or ≥ 1000/µL) is reached after the neutrophil or platelet count has bottomed out after the completion of CPX-351 administration. (iii) The recovery date is defined as the day when the platelet count (≥ 50,000/μL or ≥ 100,000/μL) or neutrophil count (≥ 500/μL or ≥ 1000/μL) is reached after the neutrophil or platelet count becomes nadir following the completion of CPX-351 administration. However, if blood cell recovery is observed during the first induction therapy, but not during the second induction therapy, the date of recovery of neutrophils or platelets during the first induction therapy is used as the evaluation date. (iv) If nadir value is higher than the above specified values, the subject patient is determined to be not recovering. (v) The following were defined as censoring: end of treatment period, death, withdrawal of patient's consent, and loss of follow-up.

2. Days of FN/pneumonia recovery

The distribution of days to febrile neutropenia or pneumonia recovery was estimated using the Kaplan–Meier method, and the median and first and third quartiles are shown. The time to recovery of febrile neutropenia or pneumonia was calculated from the start date of the first treatment day. The following were defined as censoring: end of treatment period, death, withdrawal of patient's consent, and loss of follow-up.

## Results

### Patient characteristics

This analysis included 47 patients who were included in the safety analysis set (SAF) of the Japanese P1/2 study and 153 patients who received CPX-351 and included in the Intention-to-treat analysis (ITT) of the global P3 study. In the Japanese study, there were 4.3% (*n* = 2) of t-AML compared to 19.6% (*n* = 30) in the global study, and AML from MDS with prior HMA treatment was 10.6% (*n* = 5) in Japan compared to 32.7% (*n* = 50) in the global study; AML from MDS without prior HMA treatment was 74.5% (*n* = 35) in Japan compared to 13.7% (*n* = 21) in the global study. The number of cases with platelets less than 50,000/μL at baseline was 47% (*n* = 22) in the Japanese study, and 62% (*n* = 95) in the global study (Table 1). The CR/CRi rate in the first cycle of CPX-351was 51.1% in the Japanese study and 37.9% in the global study.

**Table 1:** Patient characteristics in the Japanese P1/2 study and global P3 study

Characteristic	Japanese P1/2 study	Global P3 study
	No. (%)	No. (%)
No. of patients	47	153
Sex		
Male	31 (66.0)	94 (61.4)
Female	16 (34.0)	59 (38.6)
Age		
Median (range)	68.0 (60–75)	67.8 (60–75)
60–69	28 (59.6)	96 (62.7)
70–75	19 (40.4)	57 (37.3)
AML subtype		
Therapy-related AML	2 (4.3)	30 (19.6)
AML with antecedent MDS With prior HMA	5 (10.6)	50 (32.7)
AML with antecedent MDS Without prior *HMA*	35 (74.5)	21 (13.7)
De novo AML with MDS karyotype	4 (8.5)	41 (26.8)
AML with antecedent CMML	1 (2.1)	11 (7.2)
ECOG PS		
0	24 (51.1)	37 (24.2)
1	20 (42.6)	101 (66.0)
2	3 (6.4)	15 (9.8)
Cytogenetic risk by NCCN		
Favorable	0	7 (4.9)
Intermediate	23 (48.9)	64 (44.8)
Unfavorable	24 (51.1)	72 (50.3)
Median bone marrow blasts (aspirate), % (range)	32.6 (20.0–96.0)	35.0 (5–93)
No. with WBC count		
< 20 × 10^3^/μL	42 (89)	131 (86)
≥ 20 × 10^3^/μL	5 (11)	22 (14)
No. with PLT count		
≤ 50 × 10^3^/μL	22 (47)	95 (62)
> 50 × 10^3^/μL	25 (53)	58 (38)
No. with hemoglobin		
≤ 9 g/dL	35 (74)	100 (65)
> 9 g/dL	12 (26)	53 (35)
Treatment number of induction		
1 cycle	47 (100)	153 (100)
2 cycles	8 (17.0)	48 (31.4)
Median number of days from first treatment to 2nd induction, days (range)	34.5 (32.25–35)	18 (16–23.5)
Treatment number of consolidation		
1 cycle	25 (53.2)	49 (32.0)
2 cycles	16 (34.0)	23 (15.0)

*AML* acute myeloid leukemia, *MDS* myelodysplastic syndrome, *CMML* chronic myelomonocytic leukemia, *HMA* hypomethylating agent, *ECOG PS* Eastern Cooperative Oncology Group performance status, *NCCN* National Comprehensive Cancer Network, *WBC* white blood cell, *PLT* platelet

### Adverse events

**Fig. 1:**
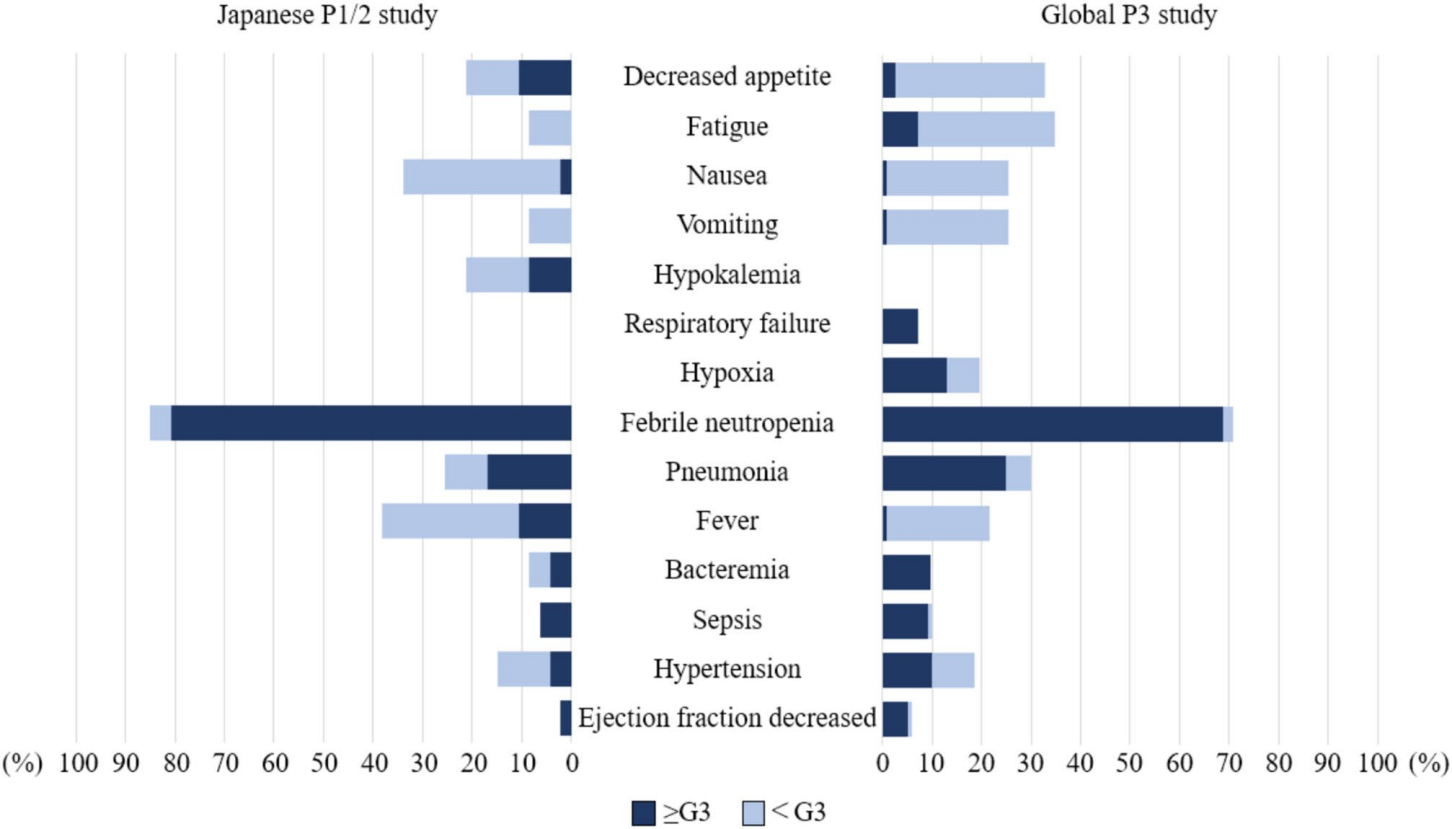
Most frequent AEs in the Japanese P1/2 study and Global P3 study. *≥ G3* grade 3 to 5; *< G3* grade 1 to 2

The most frequently observed adverse event in both the Japanese and the global studies was febrile neutropenia (85.1% in the Japanese study and 70% in the global study), and pneumonia (25.5% in the Japanese study and 30.1% in the global study) (Table 2). The adverse events identified in the Japanese study were hypokalemia (21.3%), and those identified in the global study were respiratory failure (7.2%) and hypoxia (19.6%) (Fig. 1). Skin disorder was occurred in 38 (80.9%; 4 was G3) in the Japanese study and 124 (81.0%; 14 was G3) in the global study (Supplemental table S2). Of these cases, systemic administration of corticosteroids was required in 4 cases in the Japanese study, and 7 cases in the global study. Most of the other skin disorders could be managed with topical steroids alone. Two cases were also reported as cytarabine syndrome in Japan.

**Table 2:** Most frequent AEs in the Japanese P1/2 study and global P3 study

System organ class/preferred term	Japanese P1/2 study (*N* = 47)		Global P3 study (*N* = 153)	
	All grade	≥ G3	All grade	≥ G3
	No. (%)	No. (%)	No. (%)	No. (%)
Systemic symptoms				
Decreased appetite	10 (21.3)	5 (10.6)	50 (32.7)	4 (2.6)
Fatigue	4 (8.5)	0	53 (35.0)	11 (7.2)
Nausea	16 (34.0)	1 (2.1)	75 (49.0)	1 (0.7)
Vomiting	4 (8.5)	0	39 (25.5)	1 (0.7)
Hypokalemia	10 (21.3)	4 (8.5)	0	0
Respiratory failure	0	0	11 (7.2)	11 (7.2)
Hypoxia	0	0	30 (19.6)	20 (13.0)
Infections				
Febrile neutropenia	40 (85.1)	38 (80.9)	107 (70.0)	104 (68.8)
Pneumonia	12 (25.5)	8 (17.0)	46 (30.1)	38 (24.8)
Fever	18 (38.3)	5 (10.6)	33 (21.6)	1 (0.7)
Bacteremia	4 (8.6)	2 (4.3)	15 (9.8)	15 (9.8)
Sepsis	3 (6.3)	3 (6.3)	15 (9.8)	14 (9.2)
Cardiovascular disorders				
Hypertension	7 (14.9)	2 (4.3)	29 (19.0)	16 (10.0)
Ejection fraction decreased	1 (2.1)	1 (2.1)	9 (5.9)	8 (5.2)

### Time to platelets and neutrophils recovery from induction treatment

**Fig. 2:**
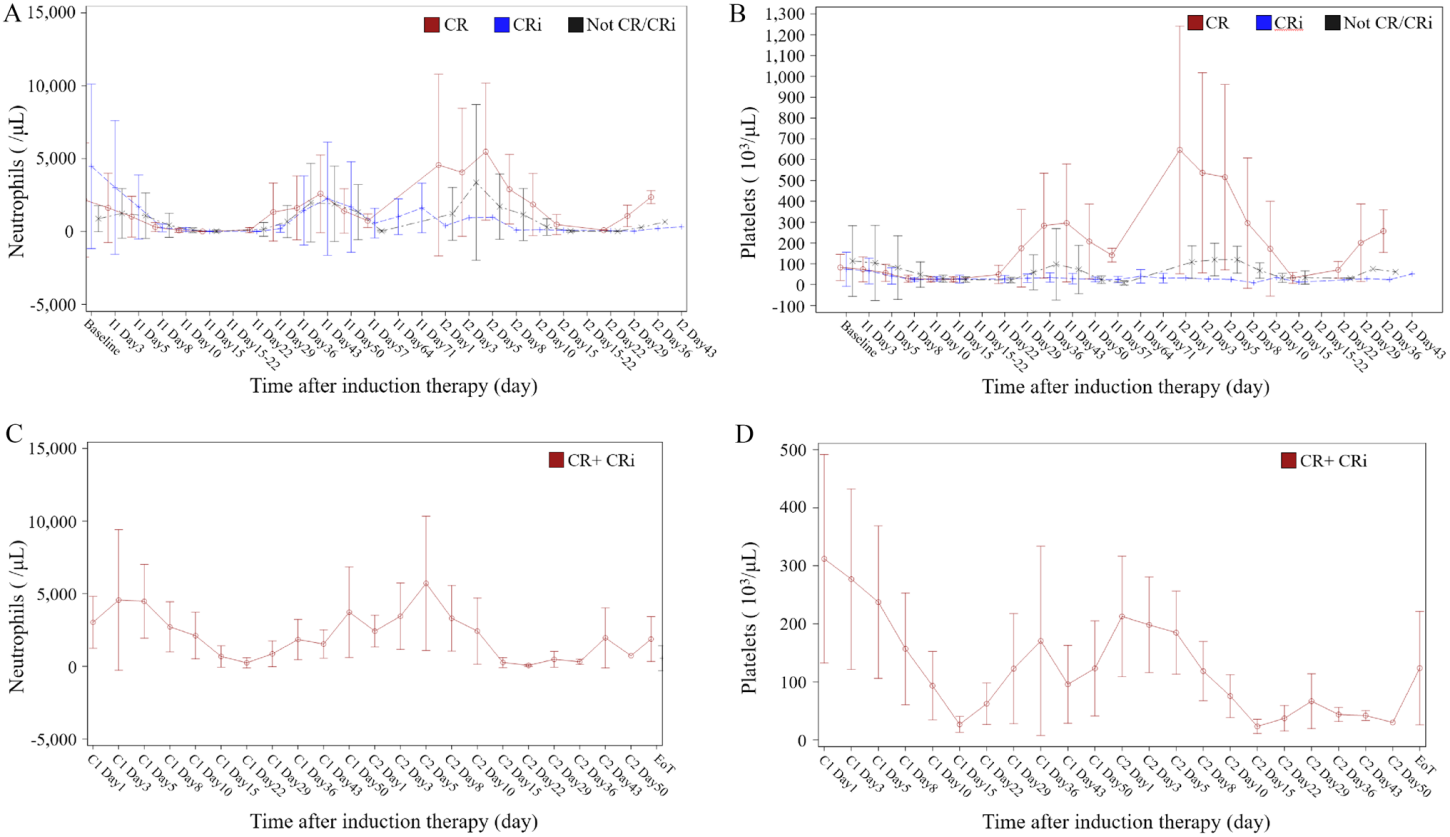
Neutrophils and platelets counts during treatment in Japanese P1/2 study. **A** Change of neutrophils during induction therapy. **B** Change of platelets during induction therapy. **C** Change of Neutrophils during consolidation therapy. **D** Change of platelets during consolidation therapy. *I1* induction cycle 1, *I2* induction cycle 2, *C1* consolidation cycle 1, *C2* consolidation cycle 2, *EoT* end of treatment, *CR* complete remission, *CRi* complete remission with incomplete blood count recovery

The changes in platelet and neutrophil counts during the induction and consolidation therapy in the Japanese study were shown in Fig. 2. For all CPX-351-treated patients, the median time to platelets recovery to 50,000/μL and 100,000/μL were 36 days (interquartile range (IQR) 29–64) and 36 days (IQR 29–73), and the median time to neutrophils recovery to 500/μL and 1000/μL were 36 days (IQR 29–43) and 36 days (IQR 31–51) in the Japanese study respectively. In the global study, the median time to platelets recovery to 50,000/µL and 100,000/µL were 37 days (IQR 34–54) and 44 days (IQR 36-NA) and the median time to neutrophils recovery to 500/µL and 1000/µL were 36 days (IQR 29–48) and 41 days (IQR 34–53) (Table S3). On the other hand, among patients who achieved CR or CRi in the first cycle of CPX-351, the median time to platelet recovery to 50,000/µL and 100,000/µL was 36 days (IQR 29–47) and 36 days (IQR 29–55) in the Japanese study and 37 days (IQR 34–44) and 42.5 days (IQR 35–49) in the global study. Similarly, for CR/CRi patients, the median time to recovery of neutrophils to 500/µL and 1,000/µL was 36 (IQR 29–47) and 36.5 (IQR: 31.0–47.0) days and 35 (IQR 29–41) and 38 (IQR 34–43) days, respectively (Table S3).

The changes in platelet and neutrophil counts during induction therapy in the Japanese cohort showed similar trends in neutrophil counts in both CR/CRi and non-CRi cases, but platelet counts in non-CRi cases were delayed compared to CR cases. To examine factors delaying neutrophil and platelet recovery, a univariate analysis was performed on the population that achieved CR/CRi after one cycle of induction therapy in the global cohort, but no factors delaying neutrophil and platelet recovery were identified (Supplemental Table S1).

### Time to FN and pneumonia occurrence

**Fig. 3:**
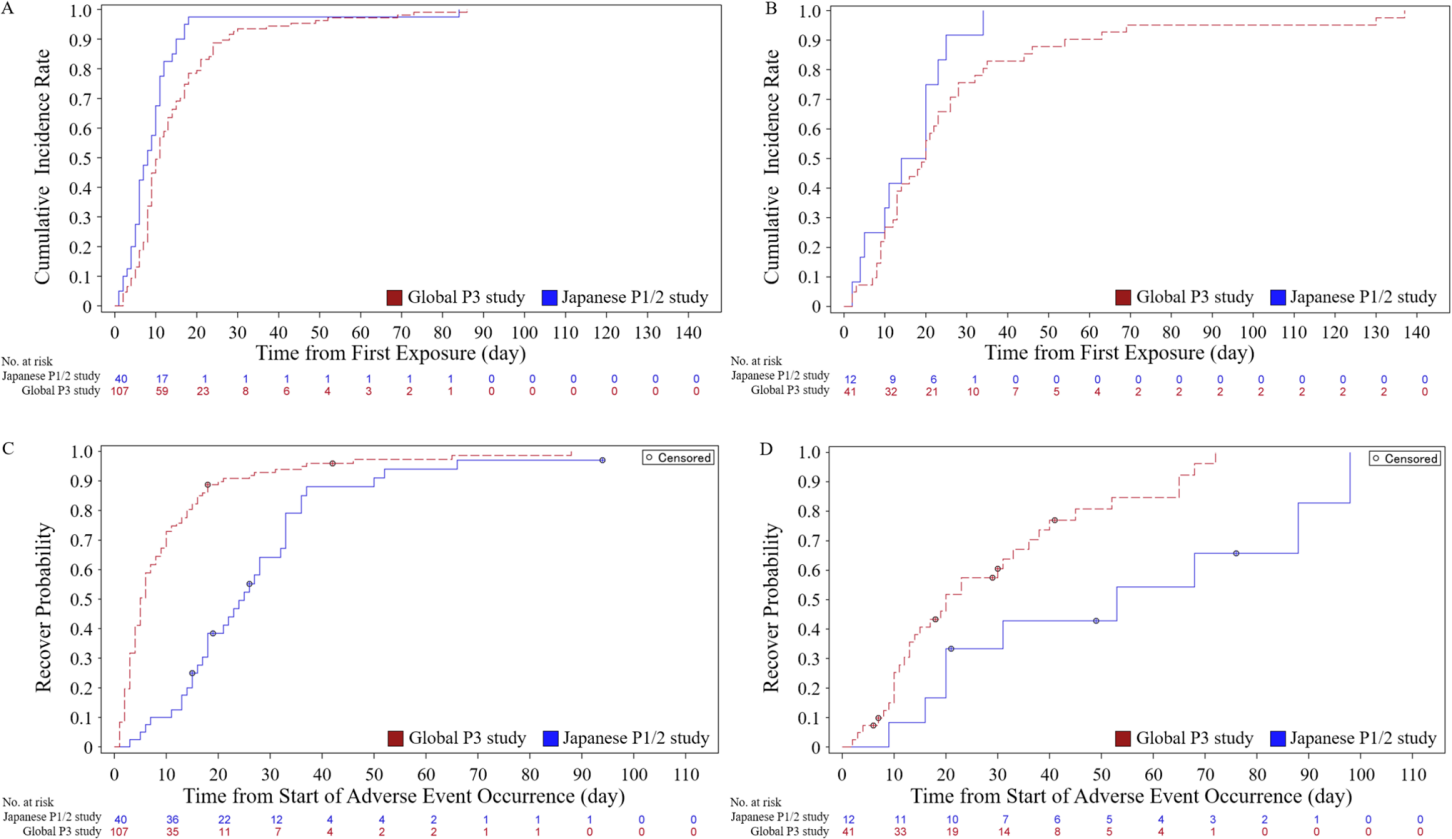
Occurrence and recovery from febrile neutropenia and pneumonia in Japanese P1/2 study and global P3 study. **A** Occurrence of febrile neutropenia in the Japanese P1/2 study and global P3 study. **B** Occurrence of pneumonia in the Japanese P1/2 study and global P3 study. **C** Recovery from febrile neutropenia in the Japanese P1/2 study and global P3 study. D Recovery from pneumonia in the Japanese P1/2 study and global P3 study

The median time to FN occurrence was 8 days in the Japanese study and 11 days in the global study (Fig. 3A), and the median time to pneumonia occurrence was 17 days and 20 days, respectively (Fig. 3B). The median time to recovery from FN was 25 days in the Japanese study and 5 days in the global study (Fig. 3C), and the median time to recovery from pneumonia was 53 days and 20 days, respectively (Fig. 3D), with a tendency toward longer periods in Japanese study. Regarding the use of G-CSF, 25.5% (*n* = 12) in Japan and 33.3% (*n* = 51) in the global study received G-CSF. Most of the G-CSFs used were short-acting, while 6 (3.9%) global patients received pegfilgrastim. In the Japanese study, the main reason for G-CSF use was for leukopenia/neutropenia in 9 cases, for treatment of pneumonia in 2 cases, and for bacteremia in 1 case. Three of the patients were administered G-CSF in order to proceed to the next course.

### Infection prophylaxis

The number of patients who received infection prophylaxis during the initial induction therapy period is shown (Table 3). In the Japanese study, 43 patients (91.5%) received infection prophylaxis, of which 40 patients (85.1%) received antibiotic prophylaxis, with levofloxacin (*n* = 20, 42.6%) the most commonly used. In addition, 13 patients (27.7%) received combination therapy with sulfonamide and trimethoprim. For fungal prophylaxis, 43 patients (91.5%) received antifungals, with fluconazole (*n* = 24, 55.8%) the most frequently used. Only 2 patients (4.3%) received acyclovir.

**Table 3:** Infection prophylaxis in the Japanese P1/2 study and global P3 study

	Japanese P1/2 study	Global P3 study
	No. (%)	No. (%)
Anti-biotic agents	40 (85.1)	100 (65.4)
Fluoroquinolones	21 (44.6)	52 (34.0)
Levofloxacin	20 (42.6)	24 (16.3)
Ciprofloxacin	1 (2.1)	24 (16.3)
Moxifloxacin	0	4 (2.6)
Third-generation cephalospolins	2 (4.3)	5 (3.3)
Cefdinir	0	2 (1.3)
Ceftazidime	1 (2.1)	1 (0.7)
Ceftriaxone	1 (2.1)	2 (1.3)
Fourth-generation cephalospolins	10 (21.3)	29 (19.0)
Cefepime	10 (21.3)	29 (19.0)
Carbapenems	2 (4.3)	5 (3.3)
Meropenem	2 (4.3)	5 (3.3)
Glycopeptide	0	17 (11.1)
Vancomycin	0	17 (11.1)
Penicillins	1 (2.1)	16 (10.5)
Piperacillin W/tazobactam	1 (2.1)	11 (7.2)
Amoxi-clavulanico	0	3 (2.0)
Amoxicillin	0	1 (0.7)
Piperacillin	0	1 (0.7)
Combinations of sulfonamides and trimethoprim	13 (27.7)	2 (1.3)
Other	2 (4.3)	24 (15.7)
Anti-fungal agents	43 (91.5)	89 (58.2)
Triazole derivatives	32 (68.1)	55 (35.9)
Fluconazole	24 (51.1)	23 (15.0)
Itraconazole	7 (14.9)	1 (0.7)
Posaconazole	0	22 (14.4)
Voriconazole	1 (2.1)	10 (6.5)
Echinocandins	8 (17.0)	30 (19.6)
Anidulafungin	0	1 (0.7)
Caspofungin	5 (10.6)	5 (3.3)
Micafungin	3 (6.4)	24 (15.7)
Polyene antimycotic	3 (6.4)	14 (9.2)
Amphotericin B	2 (4.3)	0
Amphotericin B, liposome	1 (2.1)	4 (2.6)
Nystatin	0	10 (6.5)
Anti-viral agents	2 (4.3)	99 (64.7)
Aciclovir	2 (4.3)	78 (51.0)
Famciclovir	0	1 (0.7)
Valaciclovir	0	22 (14.4)
Valganciclovir	0	1 (0.7)

On the other hand, in the global study, 129 (84.3%) received infection prophylaxis, of which 100 patients (65.4%) received antibiotic prophylaxis, most frequently with cefepime (*n* = 29, 19.0%), followed by levofloxacin (*n* = 24, 16.3%) and ciprofloxacin (*n* = 24, 16.3%). Only 2 patients (1.3%) received combination therapy with sulfonamide and trimethoprim, while 89 patients (58.2%) received antifungal prophylaxis and 99 patients (64.7%) received antiviral prophylaxis (Table 3).

### Gastrointestinal adverse events and emetic prophylaxis

In the Japanese study, nausea occurred in 16 (34.0%; one was G3), while in the global study, nausea occurred in 75 (49.0%; one was G3). Vomiting occurred in 4 (8.5%; all were G1 or 2) patients in the Japanese study and 39 (25.5%; one was G3) in the global study (Fig. 1, Table 2).

In the Japanese study, all cases received anti-emetic prophylaxis, with 45 (95.7%) receiving a 5-HT_3_ receptor antagonist (5-HT_3_-RA) and 2 (4.3%) receiving dexamethasone only or combination of neurokinin 1 receptor antagonist (NK_1_-RA) and dexamethasone. Of the cases in which 5-HT_3_-RA were used, 24 (51.1%) were used 5-HT_3_-RA alone, 12 (25.5%) with NK_1_-RA, 4 (8.5%) with dexamethasone, and 4 (8.5%) were given in combination with triplet (5-HT_3_-RA, NK_1_-RA and dexamethasone). Aprepitant was administered on consecutive days 1–5 in 10 cases (71.4%), on days 1–3 in 3 cases (21.4%), and on day 1 only in 1 case (7.1%). In the global study, 127 (83.0%) received anti-emetic prophylaxis. Of the cases in which 5-HT_3_-RA were used, 40 (26.1%) were received 5-HT_3_-RA alone, 65 (42.5%) were received 5-HT_3_-RA and dexamethasone. No cases received NK_1_-RA (Table 4).

**Table 4:** Anti-emetic prophylaxis in the Japanese P1/2 study and global P3 study

	Japanese P1/2 study	Global P3 study
	No. (%)	No. (%)
5-HT_3_ receptor antagonist	45 (95.7)	127 (83.0)
Granisetron	38 (80.9)	3 (2.0)
Ondansetron	0	124 (81.0)
Palonosetron	5 (10.6)	1 (0.7)
Ramosetron	3 (6.4)	0
NK_1_ receptor antagonist	17 (36.2)	0
Aprepitant	14 (29.8)	0
Fosaprepitant	3 (6.4)	0
Dexamethasone	10 (21.3)	78 (51.0)
Other	3 (6.4)	22 (14.4)
Prochlorperazine	0	17 (11.1)
Promethazine	0	1 (0.7)
Metoclopramide	3 (6.4)	4 (2.6)
Nabilone	0	1 (0.7)
The combination of anti-emetic agents		
5-HT_3_-RA	24 (51.1)	40 (26.1)
5-HT_3_-RA + NK_1_-RA	12 (25.5)	0
5-HT_3_-RA + DEX	4 (8.5)	65 (42.5)
5-HT_3_-RA + DEX + Other	0	11 (7.2)
5-HT_3_-RA + Other	1 (2.1)	11 (7.2)
5-HT_3_-RA + NK_1_-RA + DEX	4 (8.5)	0
NK_1_-RA + DEX	1 (2.1)	0
DEX	1 (2.1)	2 (1.3)
No anti-emetic agents	0	24 (15.7)

*5-HT*_*3*_*-RA* selective serotonin receptor antagonist, *NK*_*1*_*-RA* neurokinin 1 receptor antagonist, *DEX* dexamethasone

### Change of LVEF during CPX-351 treatment in Japanese P1/2 study

**Fig. 4:**
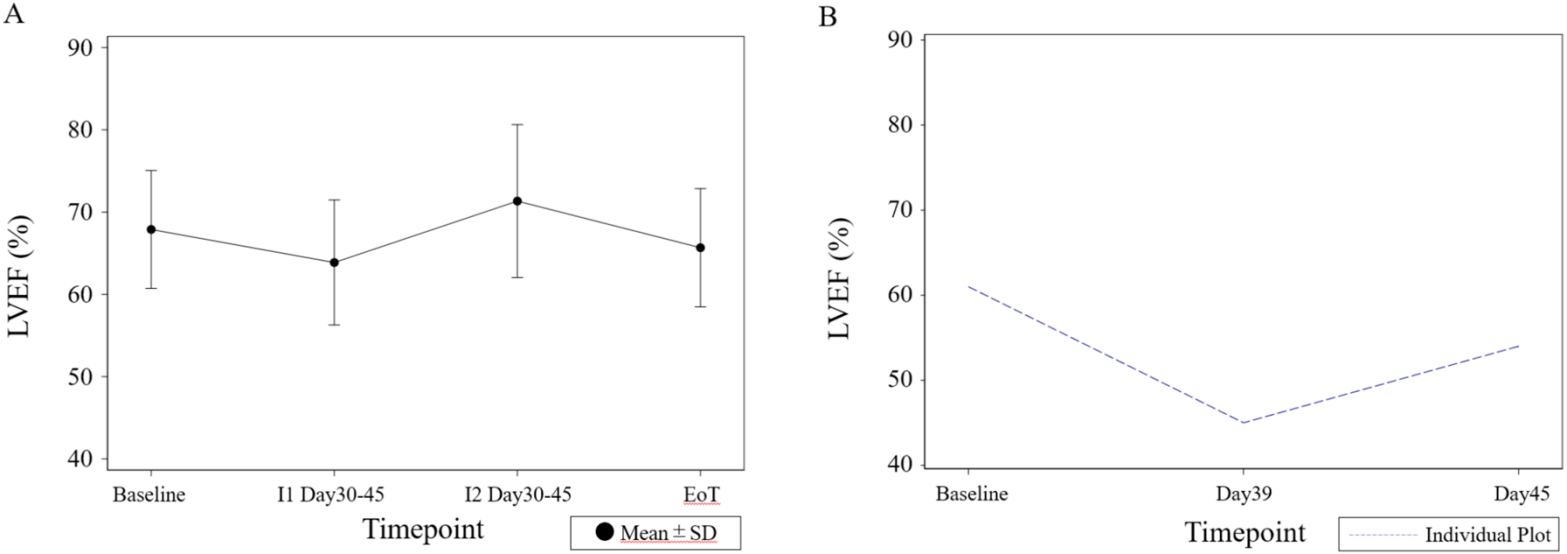
Change in LVEF during CPX-351 treatment in Japanese P1/2 study. **A** Change in LVEF during CPX-351 treatment in all Japanese P1/2 study. **B** Change in LVEF in a patient with decreased LVEF during CPX-351 treatment in Japanese P1/2 study. *I1* induction cycle 1, *I2* induction cycle 2, *EoT* end of treatment

Changes in LVEF during the treatment period are shown in Fig. 4; There were no cases in which CPX-351 treatment intervention resulted in a sudden decrease in LVEF; however, one (4.8%) patient had an LVEF < 50% at least once during the treatment period. This patient had an LVEF of 45% on day 39 of the first cycle of remission induction therapy, but there was no evidence of cardiac disease, and the LVEF recovered to 54% at the day 45 measurement (Fig. 4).

## Discussion

The adverse events of CPX-351 and 7 + 3 therapy were reported to be similar, although the time from myelosuppression to recovery was more prolonged in CPX-351 [10]. In this analysis, we investigated adverse events during CPX-351 treatment to identify differences in pattern and frequency between Japanese and non-Japanese patients.

We showed that the adverse event pattern differed between the two groups in that hypokalemia was observed only in the Japanese P1/2 study, and respiratory failure and hypoxia were observed only in the global P3 study (Table 2 and Fig. 1). However, hypokalemia was observed in 15.3% of patients treated with CPX-351 in the P2 study in untreated elderly adults with AML [15], and respiratory failure due to pneumonia or sepsis was observed in two patients in the Japanese P1/2 study. The frequency of skin disorders did not differ between the Japanese and global cohorts, and grade 3 skin disorders were observed in about 10% in both cohorts, but all were manageable. These results suggest that there is no racial difference in the incidence of adverse events, although careful observation is necessary.

For the purpose of clarifying differences in hematotoxicity between Japanese and non-Japanese, we investigated the number of days to recovery of platelets/neutrophils (Table S3). Although recovery of neutrophils and platelets was a few days longer in the global P3 study than in the Japanese P1/2 study, the recovery was similar in both groups. As for AML subtypes, there was also no difference in the number of days to platelets/neutrophils recovery between AML-MRC and t-AML. The Japanese study included only two cases of t-AML, and future studies with a large number of patients are warranted. In addition, the blood cell counts of each response to CPX-351 in the Japanese P1/2 study show that neutrophils responded well even in patients with CRi, but platelets did not (Fig. 2). Therefore, careful attention should be paid to the control of platelet counts. A univariate analysis in the global P3 study population was performed to identify the characteristics of prolonged thrombocytopenia and neutropenia, but no factors could be identified. Rondoni et al. [16] explored factors for delayed blood cell recovery in 130 patients treated with CPX-351 and found that blood cell recovery was delayed in AML that progressed from hypoplastic MDS with poor prognostic chromosomal risk. The characteristics of patients prone to prolonged myelosuppression are not well understood, further investigation is needed.

The incidence and timing of FN and pneumonia was similar in the Japanese P1/2 and global P3 studies (Fig. 1, 3). According to the French RWD, FN was frequently observed in 91% (all grade 3 or higher) and pneumonia in 36% (G3 or higher: 30%) [12], while in the German RWD, FN in grades 3–4 was observed in 15% and pneumonia in 22% [13], and in the British RWD, FN in grades 3–5 was observed in 38.1% and pneumonia in 8.2% [14]. On the other hand, in a report by the SEIFEM group on CPX-351 treatment, FN developed in 62 of 200 patients (31%) during the remission induction therapy and in 25 of 118 patients (45%) during the consolidation therapy. The incidence of FN tends to be higher during consolidation therapy, suggesting the importance of neutrophil count control during consolidation therapy [17]. In both study, G-CSF administration was permitted in cases of severe infections such as pneumonia and sepsis, with or without residual blasts. Blast clearance was confirmed in 7 of the 12 patients (58.3%) who received G-CSF in the Japanese study, and in 36 of the 48 patients (75.0%) in the global study. No statistically significant difference in overall survival (OS) was observed with or without G-CSF administration. Thus, if there is no recovery of blood cells or evidence of infection such as pneumonia, administration of G-CSF without blast clearance confirmation is a plausible option. As for the time to recovery from FN and pneumonia, it was longer in Japanese than non-Japanese patients while there is no difference in the duration of neutrophils recovery from nadir and the onset of FN and pneumonia between Japanese and non-Japanese patients. The longer recovery period for FN or pneumonia for the Japanese may be attributed to differences in the medical environment in Japan, such as the continuation of antibiotics until CRP-negative. In fact, the median duration of antibiotics treatments in the Japanese study was 23 days for FN and 43 days for pneumonia, compared with 5 days for FN and 17 days for pneumonia in the global study. In addition, the median length of hospitalization was 79 days in the Japanese P1/2 study and 41 days in the global P3 study, and these differences in hospital stay may also reflect differences in the medical environment. During CPX-351 treatment, recovery from neutropenia takes approximately 5 weeks, so infection control measures are important.

With regard to infection prophylaxis, 91.5% of patients in the Japanese P1/2 study and 84.3% of patients in the global P3 study received infection prophylaxis. Interestingly, the composition is different between the Japanese P1/2 study and the global P3 study. In the Japanese P1/2 study, anti-fungal prophylaxis was most frequently (91.5%) and anti-biotic prophylaxis was following (80.9%). However, prophylactic administration of anti-viral agents was only 4.3%. In contrast, in the global P3 study, anti-viral prophylaxis was most commonly administered at 64.7%, while anti-biotics and anti-fungals were administered only at 65.4% and 58.2%, respectively (Table 3). Although we were unable to prove a causal relationship between infection prevention and death, these results may provide useful insights into management in daily practice.

The current analysis also examined the prophylactic administration of antiemetic agents as supportive care for gastrointestinal adverse event (Table 4). Nausea was 34.0% (2.1% for G3 and above) and vomiting was 8.5% (none for G3 and above) in the Japanese P1/2 study [11], while in the global P3 study nausea was 49.0% (0.7% for G3 and above) and vomiting was 25.5% (0.7% for G3 and above) [10]. This may be due to superior management with antiemetic agents in the Japanese P1/2 study. This is because the percentage of patients who received antiemetic prophylaxis was 100% in the Japanese P1/2 trial, compared to 84.3% in the global P3 trial.

CPX-351 contains daunorubicin, and it is necessary to be careful about its cardiotoxicity. In the Japanese P1/2 study, cardiac function was evaluated by ECG as well as echocardiography, and no abnormalities were observed during the treatment period, but there was one case in which LVEF temporarily below 50% after CPX-351 administration and subsequently recovered nevertheless this patient did not have any notable cardiotoxicity risks and had not received any anthracyclines (Fig. 4). In a post hoc analysis of cardiotoxicity in the global P3 study, it was reported that LVEF and global longitudinal strain (GLS) were lower in the CPX-351 group than in the 7 + 3 group [18]. On the other hand, in the phase 1/2 Children's Oncology Group study (AAML1421), 25 patients previously treated with anthracyclines were evaluated for cardiac function, and while NT-proBNP did not change with CPX-351 treatment, there was a significant decrease in LVEF and an increase in high sensitivity troponin [19]. Although CPX-351 is liposomal formulation and is expected to reduce cardiotoxicity, it requires further investigation of its effects on cardiotoxicity.

In conclusion, the frequency and pattern of adverse events occurring during CPX-351 treatment were similar in the Japanese P1/2 and global P3 studies, suggesting that there are no racial differences in adverse events during CPX-351 treatment and the global dosage and administration is also appropriate for Japanese. There are regional differences in the type and frequency of supportive care, and patients receiving CPX-351 treatment may benefit from supportive care according to their individual characteristics.

## Limitations

This study describes adverse events in different populations and statistical comparisons between the two groups were not performed and the number of cases in the Japanese P1/2 study was small. It is necessary to interpret with caution the direct comparison of the figures of the two groups.

## Supplementary Information

Below is the link to the electronic supplementary material.

Supplementary file1 (DOCX 25 KB)

## Data Availability

All data generated or analyzed during this study are included in this published article and its supplementary information files.

## References

[1] Shallis RM, Wang R, Davidoff A, Ma X, Zeidan AM. Epidemiology of acute myeloid leukemia: recent progress and enduring challenges. Blood Rev. 2019;36:70–87. 31101526 10.1016/j.blre.2019.04.005

[2] Yamaguchi H. Advances in pathogenesis research and challenges in treatment development for acute myeloid leukemia. Int J Hematol. 2024;120(4):414–6. 39225969 10.1007/s12185-024-03837-6

[3] Wachter F, Pikman Y. Pathophysiology of acute myeloid leukemia. Acta Haematol. 2024;147(2):229–46. 38228114 10.1159/000536152

[4] Østgård LSG, Medeiros BC, Sengeløv H, Nørgaard M, Andersen MK, Dufva IH, et al. Epidemiology and clinical significance of secondary and therapy-related acute myeloid leukemia: a national population-based cohort study. J Clin Oncol. 2015;33(31):3641–9. 26304885 10.1200/JCO.2014.60.0890

[5] Hulegårdh E, Nilsson C, Lazarevic V, Garelius H, Antunovic P, Rangert Derolf Å, et al. Characterization and prognostic features of secondary acute myeloid leukemia in a population-based setting: a report from the Swedish acute leukemia registry. Am J Hematol. 2015;90(3):208–14. 25421221 10.1002/ajh.23908

[6] Xu X-Q, Wang J-M, Gao L, Qiu H-Y, Chen Li, Jia L, et al. Characteristics of acute myeloid leukemia with myelodysplasia-related changes: a retrospective analysis in a cohort of Chinese patients. Am J Hematol. 2014;89(9):874–81. 24861848 10.1002/ajh.23772

[7] Kayser S, Döhner K, Krauter J, Köhne C-H, Horst HA, Held G, et al. The impact of therapy-related acute myeloid leukemia (AML) on outcome in 2853 adult patients with newly diagnosed AML. Blood. 2011;117(7):2137–45. 21127174 10.1182/blood-2010-08-301713

[8] Tardi P, Johnstone S, Harasym N, Xie S, Harasym T, Zisman N, et al. In vivo maintenance of synergistic cytarabine:daunorubicin ratios greatly enhances therapeutic efficacy. Leuk Res. 2009;33(1):129–39. 18676016 10.1016/j.leukres.2008.06.028

[9] Lim W-S, Tardi PG, Dos Santos N, Xie X, Fan M, Liboiron BD, et al. Leukemia-selective uptake and cytotoxicity of CPX-351, a synergistic fixed-ratio cytarabine:daunorubicin formulation, in bone marrow xenografts. Leuk Res. 2010;34(9):1214–23. 20138667 10.1016/j.leukres.2010.01.015

[10] Lancet JE, Uy GL, Cortes JE, Newell LF, Lin TL, Ritchie EK, et al. CPX-351 (cytarabine and daunorubicin) liposome for injection versus conventional cytarabine plus daunorubicin in older patients with newly diagnosed secondary acute myeloid leukemia. J Clin Oncol. 2018;36(26):2684–92. 30024784 10.1200/JCO.2017.77.6112 PMC6127025

[11] Usuki K, Miyamoto T, Yamauchi T, Ando K, Ogawa Y, Onozawa M, et al. A phase 1/2 study of NS-87/CPX-351 (cytarabine and daunorubicin liposome) in Japanese patients with high-risk acute myeloid leukemia. Int J Hematol. 2024;119(6):647–59. 38532078 10.1007/s12185-024-03733-z PMC11136735

[12] Chiche E, Rahmé R, Bertoli S, Dumas P-Y, Micol J-B, Hicheri Y, et al. Real-life experience with CPX-351 and impact on the outcome of high-risk AML patients: a multicentric French cohort. Blood Adv. 2021;5(1):176–84. 33570629 10.1182/bloodadvances.2020003159 PMC7805314

[13] Rautenberg C, Stölzel F, Röllig C, Stelljes M, Gaidzik V, Lauseker M, et al. Real-world experience of CPX-351 as first-line treatment for patients with acute myeloid leukemia. Blood Cancer J. 2021;11(10):164. 34608129 10.1038/s41408-021-00558-5 PMC8490353

[14] Mehta P, Campbell V, Maddox J, Floisand Y, Kalakonda AJM, O’Nions J, et al. CREST-UK: Real-world effectiveness, safety and outpatient delivery of CPX-351 for first-line treatment of newly diagnosed therapy-related AML and AML with myelodysplasia-related changes in the UK. Br J Haematol. 2024;205(4):1326–36. 38977430 10.1111/bjh.19622

[15] Lancet JE, Cortes JE, Hogge DE, Tallman MS, Kovacsovics TJ, Damon LE, Komrokji R, et al. Phase 2 trial of CPX-351, a fixed 5:1 molar ratio of cytarabine/daunorubicin, vs cytarabine/daunorubicin in older adults with untreated AML. Blood. 2014;123(21):3239–46. 24687088 10.1182/blood-2013-12-540971 PMC4624448

[16] Rondoni M, Minetto P, Puglisi B, Saraceni F, Brunetti L, Mianulli AM, et al. Late hematologic recovery after CPX-351 induction chemotherapy in AML: predictive disease and patient’s factors and their impact on survival. In: 65th annual meeting of the American Society of Hematlogy; December 9–12, 2023; San Diego, America. Abstract 4275.

[17] Fianchi L, Guolo F, Marchesi F, Cattaneo C, Gottardi M, Restuccia F, et al. Multicenter observational retrospective study on febrile events in patients with acute myeloid leukemia treated with Cpx-351 in “Real-Life”: the SEIFEM experience. Cancers (Basel). 2023;15(13):3457. 37444567 10.3390/cancers15133457 PMC10341225

[18] Mitchell J, Pfeiffer M, Boehmer J, Gorcsan J, Dronamraju N, Faderl S, et al. Cardiotoxicity of CPX-351 vs 7+3 in patients with untreated high-risk acute myeloid leukemia. In: 2023 American Society of Clinical Oncology (ASCO) Annual Meeting; June 2–6, 2023; Chicago, America. Abstract 7029.

[19] Leger KJ, Absalon MJ, Demissei BG, Smith AM, Gerbing RB, Alonzo TA, et al. Cardiotoxicity of CPX-351 in children and adolescents with relapsed AML: a Children’s Oncology Group report. Front Cardiovasc Med. 2024;11:1347547. 38947228 10.3389/fcvm.2024.1347547 PMC11211570

