# Supplementary material for: Safety of liposomal daunorubicin-cytarabine (CPX-351) in secondary AML: Japanese phase 1/2 study and global phase 3 study
Source: Int J Hematol. 2025 Aug 15;122(6):824–34. doi: 10.1007/s12185-025-04047-4 (PMC12638407; doi:10.1007/s12185-025-04047-4)
Supplement: Supplementary file 1 — Supplementary file1 (DOCX 25 KB) [file 12185_2025_4047_MOESM1_ESM.docx]

**Supplementary Material**

**･Title**

**Safety of liposomal daunorubicin-cytarabine (CPX-351) in secondary AML: Japanese P1/2 study and global P3 study**

**･Author names and affiliations**

Naoko Hosono^1^, Yusaku Tomiyama^2^, Nanako Emori^2^, Kento Isogaya^3^, Takahiro Yamauchi^1^

1 Department of Hematology and Oncology, Faculty of Medical Sciences, University of Fukui, Fukui, Japan

2 Medical Planning Department, Nippon Shinyaku Co., Ltd, Kyoto, Japan.

3 Data Science Department, Nippon Shinyaku Co., Ltd, Kyoto, Japan.

**･Corresponding author**

Full name: Naoko Hosono

Address: 23-3 Matsuoka Shimoaizuki, Eiheiji-cho, Yoshida-gun, Fukui 910-1193, Japan,

phone number: +81-776-61-3111, fax number: +81-776-61-8109

E-mail address: hosono@u-fukui.ac.jp

Tables

Table S1. Variables in univariate analysis for time to platelets or neutrophils recovery within the CPX-351 treated patients who achieved CR or CRi in the first induction in the global P3 study

| Age | Baseline LDH |
| --- | --- |
| Age: 60-69y.o. vs 70-75y.o. | Baseline cellularity |
| Race: Asian vs White | PB blast 20-40% vs >60% |
| Race: Other vs White | PB blast <20% vs >60% |
| Race: HISPANIC OR LATINO vs NOT HISPANIC OR LATINO | PB blast >40-60% vs >60% |
| Sex: woman vs man | PB blast 1-10% vs >10% |
| Body weight | FLT3ITD mut negative vs positive |
| Height | NPM1 mut negative vs positive |
| BSA | CEBPA mut negative vs positive |
| BMI | AML subtype |
| ECOG PS | Prior CMML vs Therapy-related AML |
| Hospitalization | De novo AML with MDS karyotype vs Therapy-related AML |
| Baseline Hb ≤9g/dL vs >9g/dL | AML with antecedent MDS with prior HMA vs Therapy-related AML |
| Baseline PLT ≤50×10^3^/L vs >50×10^3^/L | AML with antecedent MDS without prior HMA vs Therapy-related AML |
| Baseline WBC ≤20×10^3^/L vs >20×10^3^/L | Cytogenetic risk |
| Total dosage of CPX-351 | Better risk vs Poor risk |
| Total dosage of anthracycline | Intermediate risk vs Poor risk |
| Total dosage of CPX-351 in induction | Non-poor vs Poor |
| Total dosage of CPX-351 in consolidation |  |

Table S2. Skin and subcutaneous tissue disorders in the Japanese P1/2 study and global P3 study

| System Organ Class / | Japanese P1/2 study  （N=47） | Global P3 study  （N=153） |
| --- | --- | --- |
| Preferred Term | No. (%) | No. (%) |
| Skin and subcutaneous tissue disorders | 38 (80.9) | 124 (81.0) |
| Grade 1 | 13 (27.7) | 56 (36.6) |
| Grade 2 | 21 (44.3) | 54 (35.3) |
| Grade 3 | 4 (8.5) | 14 (9.2) |
| Grade 4 | 0 | 0 |
| Grade 5 | 0 | 0 |
| Grade 3 Skin and subcutaneous tissue disorders |  |  |
| Acute febrile neutrophilic dermatosis | 0 | 1 (0.7) |
| Hyperhidrosis | 0 | 1 (0.7) |
| Purpura | 0 | 1 (0.7) |
| Rash | 2 (4.3) | 4 (2.6) |
| Rash generalised | 0 | 1 (0.7) |
| Rash macular | 0 | 1 (0.7) |
| Rash maculo-papular | 1 (2.1) | 1 (0.7) |
| Rash pruritic | 1 (2.1) | 1 (0.7) |
| Skin lesion | 0 | 1 (0.7) |
| Skin ulcer | 0 | 1 (0.7) |
| Skin ulcer hemorrhage | 0 | 1 (0.7) |
| Received topical steroid | 26 (68.4) | 51 (41.1) |

Table S3. Time to platelets and neutrophils recovery in the Japanese P1/2 study and global P3 study

|  | Japanese P1/2 study | Global P3 study |
| --- | --- | --- |
|  | Median day(Q1, Q3) | Median day(Q1, Q3) |
| All, n | 47 | 153 |
| Time to platelet recovery ≥50,000 platelets/μL | 36 (29, 64) | 37 (34, 54) |
| Time to platelet recovery ≥100,000 platelets/μL | 36 (29, 73) | 44 (36, NA) |
| Time to neutrophil recovery ≥500 neutrophils/μL | 36 (29, 43) | 36 (29, 48) |
| Time to neutrophil recovery ≥1,000 neutrophils/μL | 36 (31, 51) | 41 (34, 53) |
| Achieve CR/CRi in 1 induction cycle, n | 24 | 58 |
| Time to platelet recovery ≥50,000 platelets/μL | 36 (29, 47) | 37 (34, 44) |
| Time to platelet recovery ≥100,000 platelets/μL | 36 (29, 54.5) | 42.5 (35, 49) |
| Time to neutrophil recovery ≥500 neutrophils/μL | 36 (29, 47) | 35 (29, 41) |
| Time to neutrophil recovery ≥1,000 neutrophils/μL | 36.5 (31, 47) | 38 (34, 43) |
| AML-MRC, n | 44 | 112 |
| Time to platelet recovery ≥50,000 platelets/μL | 36 (29, 64) | 37 (34, 48) |
| Time to platelet recovery ≥100,000 platelets/μL | 36 (30, 73) | 44 (36, NA) |
| Time to neutrophil recovery ≥500 neutrophils/μL | 36 (29, 50) | 35 (29, 44) |
| Time to neutrophil recovery ≥1,000 neutrophils/μL | 37 (31, 51) | 40 (34, 49) |
| Therapy-related AML, n | 2 | 30 |
| Time to platelet recovery ≥50,000 platelets/μL | 29 (22, 36) | 41 (34, 62) |
| Time to platelet recovery ≥100,000 platelets/μL | 33.5 (29, 38) | 41 (34, NA) |
| Time to neutrophil recovery ≥500 neutrophils/μL | 32.5 (29, 36) | 37 (34, 49) |
| Time to neutrophil recovery ≥1,000 neutrophils/μL | 33.5 (31, 36) | 41 (34, 55) |
